# Three Decades of Research on O-GlcNAcylation – A Major Nutrient Sensor That Regulates Signaling, Transcription and Cellular Metabolism

**DOI:** 10.3389/fendo.2014.00183

**Published:** 2014-10-27

**Authors:** Gerald W. Hart

**Affiliations:** ^1^Department of Biological Chemistry, School of Medicine, Johns Hopkins University, Baltimore, MD, USA

**Keywords:** O-GlcNAcylation, O-GlcNAc transferase, O-GlcNAcase, signaling, transcription, diabetes, cancer, Alzheimer’s disease

## Abstract

Even though the dynamic modification of polypeptides by the monosaccharide, O-linked N-acetylglucosamine (O-GlcNAcylation) was discovered over 30 years ago, its physiological significance as a major nutrient sensor that regulates myriad cellular processes has only recently been more widely appreciated. O-GlcNAcylation, either on its own or by its interplay with other post-translational modifications, such as phosphorylation, ubiquitination, and others, modulates the activities of signaling proteins, regulates most components of the transcription machinery, affects cell cycle progression and regulates the targeting/turnover or functions of myriad other regulatory proteins, in response to nutrients. Acute increases in O-GlcNAcylation protect cells from stress-induced injury, while chronic deregulation of *O*-GlcNAc cycling contributes to the etiology of major human diseases of aging, such as diabetes, cancer, and neurodegeneration. Recent advances in tools to study O-GlcNAcylation at the individual site level and specific inhibitors of *O*-GlcNAc cycling have allowed more rapid progress toward elucidating the specific functions of O-GlcNAcylation in essential cellular processes.

## Early History

O-GlcNAcylation was discovered in the early 1980s when bovine milk galactosyltransferase was used as an enzymatic probe of terminal *N*-acetylglucosamine moieties in cells of the murine immune system (1). Later studies established *O*-GlcNAc’s surprising nucleocytoplasmic subcellular localization and distribution at a time when dogma stated that protein glycosylation only occurs within the secretory pathway or extracellular compartments (2). O-GlcNAcylation was shown to be highly abundant within the nucleus and particularly enriched at the nuclear envelope and on nuclear pore proteins (3–5). However, O-GlcNAcylation was also found to be abundant on cytoskeletal proteins of human erythrocytes, which lack a nucleus (6). Viruses were also found to contain O-GlcNAcylated proteins, which occur on proteins surrounding their nucleic acid cores, rather than on their capsids, where other forms of “classical” protein glycosylation are found (7). O-GlcNAcylation was subsequently found to be highly enriched on proteins associated with chromatin in *Drosophila* (8), and *O*-GlcNAc was shown to not only be a major modifier of transcription factors (9), but also a major modification of the C-terminal domain (CTD) of RNA polymerase II itself (10). Early studies in lymphocytes showed that cellular activation resulted in rapid changes, suggesting that *O*-GlcNAc cycled like phosphorylation and could be a regulatory modification (11), which was later confirmed by the sugar’s rapid cycling on small heat shock proteins, shown by classical pulse-chase analyses (12). An assay for *O*-GlcNAc transferase (OGT), based upon tritiated UDP-GlcNAc as the donor and synthetic peptide acceptors, was developed and OGT activity was identified and characterized (13). OGT was subsequently purified to apparent homogeneity by brute-force biochemical approaches combined with nucleotide affinity chromatography (14). O-GlcNAcase was originally purified from rat spleen cytosol (15) and was found to be similar to hexosaminidase C (16, 17), which was known but had not been purified to homogeneity. Based upon polypeptide sequencing, in conjunction with PCR cloning, the *OGT* cDNA from rat (18), *C. elegans* and human (19) were cloned. OGT was found to be a very highly conserved protein with no homology to other known glycosyltransferases. OGT was also found to have two distinct domains, a catalytic domain and a protein–protein interaction domain consisting of over 11 tetratricopeptide (TPR) repeats separated by a linker region. Likewise, O-GlcNAcase was purified from bovine brain and the protein was sequenced by mass spectrometry, and used to clone the enzyme from a human library (20). The *OGA* gene was found to be identical to *MGEA5* a putative hyaluronidase associated with meningioma (21). Early studies identified *O*-GlcNAc on nuclear receptors, tau protein in the brain, intermediate filament proteins, nuclear oncogenes and tumor suppressors, and many other proteins with a wide-range of functions [reviewed in Ref. (22)].

## More Recent Findings

As the tools for the detection and analysis of O-GlcNAcylation improved, it became apparent that this post-translational modification is much more abundant than previously expected [reviewed in Ref. (23, 24)]. In addition, it was soon realized that not only was the donor for O-GlcNAcylation, UDP-GlcNAc, a major node of metabolism, but also that *O*-GlcNAc has extensive interplay with protein phosphorylation [reviewed in Ref. (23)]. Gene deletion studies have shown that both OGT and O-GlcNAcase are essential genes in mammals and plants (25–27).

Like phosphorylation and ubquitination, O-GlcNAcylation regulates many different cellular processes. O-GlcNAcylation is essential in the process of lymphocyte activation in both B- and T-lymphocytes (28). There are several examples where the glycan regulates protein:protein interactions [e.g., Ref. (29, 30)]. Nutrients fine-tune circadian clocks via O-GlcNAcylation (31–34). *O*-GlcNAc modulates the activity of the proteasome (35–39), and also has interplay with ubquitination (40, 41). Recent studies indicate that O-GlcNAcylation is very important to neuronal and brain functions, including synaptic plasticity, synaptic vesicle trafficking, and axonal branching (42–46). O-GlcNAcylation also regulates growth hormone signaling in plants (27), protects cells from acute stresses (47), and modulates transition through the cell cycle (48). Even though O-GlcNAcylation has not yet been documented to occur in yeast, such as *Saccharomyces cerevisiae* or *Schizosaccharomyces pombe*, *O*-GlcNAc does occur in some of oldest known eukaryotes (49), including in some important human parasites (50–52). In certain bacteria, O-GlcNAcylation regulates flagellar motility (53), and in *Streptococcus pneumonia*, O-GlcNAcylation of an adhesion plays a role in infection and pathogenesis (54). However, the bacterial OGT involved in each case is quite different from the eukaryotic enzyme.

As a key nutrient sensor, O-GlcNAcylation is fundamentally important to the regulation of transcription at nearly all levels, including regulation of the functions of RNA polymerase II itself (55, 56), modulating the activities of nearly all transcription factors (30), regulating both histone and DNA methylation (57–61), crosstalking with other epigenetic modifications (62), and serving as an integral part of the histone code (63). Not only does O-GlcNAcylation have extensive crosstalk with protein phosphorylation at the protein site level but also the sugar modifies many kinases and regulates their activities or specificity (64–69).

Given the myriad functions associated with O-GlcNAcylation, it is not surprising that this nutrient sensor plays a fundamental role in the etiology of diabetes and glucose toxicity (70–72). O-GlcNAcylation is elevated in all cancers studied to date and appears to play a role in tumor cell progression (24, 73, 74), and in patient prognosis (75). Given O-GlcNAcylation’s abundance and presence on hundreds of proteins in the brain, it is also a major mechanism contributing to neurodegeneration (76–78). After 30-years of research on O-GlcNAcylation, it is now not only more apparent than ever that this post-translation modification plays a central role in the nutrient regulation of cellular physiology but also it is clear that we have a long way to go to fully understand the importance of O-GlcNAcylation in most cellular and disease processes.

## Future Directions

Many important questions remain with respect to O-GlcNAcylation. (1) How does *O*-GlcNAc cycling achieve substrate specificity with only two known genes in mammals, *OGT* and *OGA (MGEA5)*? Clearly, several different mechanisms are involved. *In vitro*, OGT has remarkable specificity for peptide subtrates, which appears to change with UDP-GlcNAc concentrations (79). Most importantly, both enzymes function as part of transient holoenzyme complexes, which number in the hundreds, are cell type specific and serve to target the enzymes to their specific substrates. A key question is how is the formation of these holoenzyme complexes regulated by nutrients and other signals? (2) How are kinases regulated by O-GlcNAcylation? Many kinases are dynamically O-GlcNAcylated, and thus far, those studied are regulated by the glycan. How does this observation alter our view of signaling and system biological studies of cellular physiology? (3) How does O-GlcNAcylation play a role in neuronal functions and in learning and memory? O-GlcNAcylation is incredibly abundant in the mammalian brain, and in neurons, particularly at the synapse and in dendritic spines (42, 45, 80). Elucidation of *O*-GlcNAc’s roles in normal neuronal functions and in brain biology will become a huge area of future research. (4) What are the specific roles of O-GlcNAcylation in nutrient regulation of transcription? While it is now clear that O-GlcNAcylation is fundamentally important in nearly every aspect of transcription, we currently know almost nothing with respect to its protein-specific or site-specific roles on individual transcription regulatory proteins. This area will remain an enormous challenge for some time to come.

Finally, while the tools to study O-GlcNAcylation have advanced substantially in the last three decades, there remains an acute need to develop better methods and approaches that can be applied by biologists. These include: (1) The development of many site-specific *O*-GlcNAc antibodies; (2) A molecular biology approach to either mimic O-GlcNAcylation or to generate site-specific O-GlcNAcylation on proteins; (3) Methods are need that can raise or lower O-GlcNAcylation on individual proteins or at individual sites to evaluate functions. Unfortunately, current methods either based upon inhibitors or genetic approaches to alter O-GlcNAcylation, all act globally. (5) There continues to be a need for better methods to both detect and site-map *O*-GlcNAc on proteins. The challenges in this field are large but so is the pay off for our understanding of cellular physiology and chronic disease.

## Conflict of Interest Statement

The author declares that the research was conducted in the absence of any commercial or financial relationships that could be construed as a potential conflict of interest.

## References

[1] Torres C-R Hart GW. Topography and polypeptide distribution of terminal N-acetylglucosamine residues on the surfaces of intact lymphocytes. J Biol Chem (1984) 259:3308–17. 6421821

[2] Holt GD Hart GW. The subcellular distribution of terminal N-acetylglucosamine moieties. Localization of a novel protein-saccharide linkage, O-linked GlcNAc. J Biol Chem (1986) 261:8049–57. 3086323

[3] Hanover JA Cohen CK Willingham MC Park MK. O-linked N-acetylglucosamine is attached to proteins of the nuclear pore. Evidence for cytoplasmic and nucleoplasmic glycoproteins. J Biol Chem (1987) 262:9887–94. 3110163

[4] Davis LI Blobel G. Nuclear pore complex contains a family of glycoproteins that includes p62: glycosylation through a previously unidentified cellular pathway. Proc Natl Acad Sci U S A (1987) 84:7552–6. 10.1073/pnas.84.21.7552 3313397 PMC299337

[5] Holt GD Snow CM Senior A Haltiwanger RS Gerace L Hart GW. Nuclear pore complex glycoproteins contain cytoplasmically disposed O-linked N-acetylglucosamine. J Cell Biol (1987) 104:1157–64. 10.1083/jcb.104.5.1157 3571327 PMC2114481

[6] Holt GD Haltiwanger RS Torres CR Hart GW. Erythrocytes contain cytoplasmic glycoproteins. O-linked GlcNAc on Band 4.1. J Biol Chem (1987) 262:14847–50. 3117790

[7] Benko DM Haltiwanger RS Hart GW Gibson W. Virion basic phosphoprotein from human cytomegalovirus contains O-linked N-acetylglucosamine. Proc Natl Acad Sci U S A (1988) 85:2573–7. 10.1073/pnas.85.8.2573 2833746 PMC280039

[8] Kelly WG Hart GW. Glycosylation of chromosomal proteins: localization of O-linked N-acetylglucosamine in *Drosophila* chromatin. Cell (1989) 57:243–51. 10.1016/0092-8674(89)90962-8 2495182

[9] Jackson SP Tjian R. O-glycosylation of eukaryotic transcription factors: implications for mechanisms of transcriptional regulation. Cell (1988) 55:125–33. 10.1016/0092-8674(88)90015-3 3139301

[10] Kelly WG Dahmus ME Hart GW. RNA polymerase II is a glycoprotein. Modification of the COOH-terminal domain by O-GlcNAc. J Biol Chem (1993) 268:10416–24. 8486697

[11] Kearse KP Hart GW. Lymphocyte activation induces rapid changes in nuclear and cytoplasmic glycoproteins. Proc Natl Acad Sci U S A (1991) 88:1701–5. 10.1073/pnas.88.5.1701 2000378 PMC51092

[12] Roquemore EP Chevrier MR Cotter RJ Hart GW. Dynamic O-GlcNAcylation of the small heat shock protein alpha B-crystallin. Biochemistry (1996) 35:3578–86. 10.1021/bi951918j 8639509

[13] Haltiwanger RS Holt GD Hart GW. Enzymatic addition of O-GlcNAc to nuclear and cytoplasmic proteins. Identification of a uridine diphospho-N-acetylglucosamine:peptide beta-N-acetylglucosaminyltransferase. J Biol Chem (1990) 265:2563–8. 2137449

[14] Haltiwanger RS Blomberg MA Hart GW. Glycosylation of nuclear and cytoplasmic proteins. Purification and characterization of a uridine diphospho-N-acetylglucosamine:polypeptide beta-N-acetylglucosaminyltransferase. J Biol Chem (1992) 267:9005–13. 1533623

[15] Dong DL Hart GW. Purification and characterization of an O-GlcNAc selective N-acetyl-beta-d-glucosaminidase from rat spleen cytosol. J Biol Chem (1994) 269:19321–30. 8034696

[16] Overdijk B Van der Kroef WM Van Steijn GJ Lisman JJ. Isolation and further characterization of bovine brain hexosaminidase C. Biochim Biophys Acta (1981) 659:255–66. 10.1016/0005-2744(81)90052-8 7260095

[17] Braidman I Carroll M Dance N Robinson D Poenaru L Weber A Characterisation of human N-acetyl-beta-hexosaminidase C. FEBS Lett (1974) 41:181–4. 10.1016/0014-5793(74)81206-8 4859245

[18] Kreppel LK Blomberg MA Hart GW. Dynamic glycosylation of nuclear and cytosolic proteins. Cloning and characterization of a unique O-GlcNAc transferase with multiple tetratricopeptide repeats. J Biol Chem (1997) 272:9308–15. 10.1074/jbc.272.14.9308 9083067

[19] Lubas WA Frank DW Krause M Hanover JA. O-linked GlcNAc transferase is a conserved nucleocytoplasmic protein containing tetratricopeptide repeats. J Biol Chem (1997) 272:9316–24. 10.1074/jbc.272.14.9316 9083068

[20] Gao Y Wells L Comer FI Parker GJ Hart GW. Dynamic O-glycosylation of nuclear and cytosolic proteins: cloning and characterization of a neutral, cytosolic beta-N-acetylglucosaminidase from human brain. J Biol Chem (2001) 276:9838–45. 10.1074/jbc.M010420200 11148210

[21] Comtesse N Maldener E Meese E. Identification of a nuclear variant of MGEA5, a cytoplasmic hyaluronidase and a beta-N-acetylglucosaminidase. Biochem Biophys Res Commun (2001) 283:634–40. 10.1006/bbrc.2001.4815 11341771

[22] Hart GW. Dynamic O-linked glycosylation of nuclear and cytoskeletal proteins. Ann Rev Biochem (1997) 66:315–35. 10.1146/annurev.biochem.66.1.315 9242909

[23] Hart GW Slawson C Ramirez-Correa G Lagerlof O. Cross talk between O-GlcNAcylation and phosphorylation: roles in signaling, transcription, and chronic disease. Annu Rev Biochem (2011) 80:825–58. 10.1146/annurev-biochem-060608-102511 21391816 PMC3294376

[24] Hardiville S Hart GW. Nutrient regulation of signaling, transcription, and cell physiology by O-GlcNAcylation. Cell Metab (2014) 20:208–13. 10.1016/j.cmet.2014.07.014 25100062 PMC4159757

[25] Shafi R Iyer SP Ellies LG O’Donnell N Marek KW Chui D The O-GlcNAc transferase gene resides on the X chromosome and is essential for embryonic stem cell viability and mouse ontogeny. Proc Natl Acad Sci U S A (2000) 97:5735–9. 10.1073/pnas.100471497 10801981 PMC18502

[26] Hartweck LM Scott CL Olszewski NE. Two O-linked N-acetylglucosamine transferase genes of *Arabidopsis thaliana* L. Heynh. have overlapping functions necessary for gamete and seed development. Genetics (2002) 161:1279–91. 12136030 10.1093/genetics/161.3.1279 PMC1462182

[27] Olszewski NE West CM Sassi SO Hartweck LM. O-GlcNAc protein modification in plants: evolution and function. Biochim Biophys Acta (2010) 1800:49–56. 10.1016/j.bbagen.2009.11.016 19961900 PMC2815191

[28] Golks A Tran TT Goetschy JF Guerini D. Requirement for O-linked N-acetylglucosaminyltransferase in lymphocytes activation. EMBO J (2007) 26:4368–79. 10.1038/sj.emboj.7601845 17882263 PMC2034663

[29] Hiromura M Choi CH Sabourin NA Jones H Bachvarov D Usheva A. YY1 is regulated by O-linked N-acetylglucosaminylation (O-glcNAcylation). J Biol Chem (2003) 278:14046–52. 10.1074/jbc.M300789200 12588874

[30] Ozcan S Andrali SS Cantrell JE. Modulation of transcription factor function by O-GlcNAc modification. Biochim Biophys Acta (2010) 1799:353–64. 10.1016/j.bbagrm.2010.02.005 20202486 PMC2881704

[31] Durgan DJ Pat BM Laczy B Bradley JA Tsai JY Grenett MH O-GlcNAcylation, novel post-translational modification linking myocardial metabolism and cardiomyocyte circadian clock. J Biol Chem (2011) 286:44606–19. 10.1074/jbc.M111.278903 22069332 PMC3247942

[32] Kim EY Jeong EH Park S Jeong HJ Edery I Cho JW. A role for O-GlcNAcylation in setting circadian clock speed. Genes Dev (2012) 26:490–502. 10.1101/gad.182378.111 22327476 PMC3305986

[33] Kaasik K Kivimae S Allen JJ Chalkley RJ Huang Y Baer K Glucose sensor O-GlcNAcylation coordinates with phosphorylation to regulate circadian clock. Cell Metab (2013) 17:291–302. 10.1016/j.cmet.2012.12.017 23395175 PMC3597447

[34] Hart GW. How sugar tunes your clock. Cell Metab (2013) 17:155–6. 10.1016/j.cmet.2013.01.008 23395163 PMC3636988

[35] Sumegi M Hunyadi-Gulyas E Medzihradszky KF Udvardy A. 26S proteasome subunits are O-linked N-acetylglucosamine-modified in *Drosophila melanogaster*. Biochem Biophys Res Commun (2003) 312:1284–9. 10.1016/j.bbrc.2003.11.074 14652013

[36] Liu K Paterson AJ Zhang F McAndrew J Fukuchi K Wyss JM Accumulation of protein O-GlcNAc modification inhibits proteasomes in the brain and coincides with neuronal apoptosis in brain areas with high O-GlcNAc metabolism. J Neurochem (2004) 89:1044–55. 10.1111/j.1471-4159.2004.02389.x 15140202

[37] Kudlow JE. Post-translational modification by O-GlcNAc: another way to change protein function. J Cell Biochem (2006) 98:1062–75. 10.1002/jcb.20926 16598783

[38] Bowe DB Sadlonova A Toleman CA Novak Z Hu Y Huang P O-GlcNAc integrates the proteasome and transcriptome to regulate nuclear hormone receptors. Mol Cell Biol (2006) 26:8539–50. 10.1128/MCB.01053-06 16966374 PMC1636782

[39] Zhang F Su K Yang X Bowe DB Paterson AJ Kudlow JE. O-GlcNAc modification is an endogenous inhibitor of the proteasome. Cell (2003) 115:715–25. 10.1016/S0092-8674(03)00974-7 14675536

[40] Guinez C Mir AM Dehennaut V Cacan R Harduin-Lepers A Michalski JC Protein ubiquitination is modulated by O-GlcNAc glycosylation. FASEB J (2008) 22:2901–11. 10.1096/fj.07-102509 18434435

[41] Ruan HB Nie Y Yang X. Regulation of protein degradation by O-GlcNAcylation: crosstalk with ubiquitination. Mol Cell Proteomics (2013) 12:3489–97. 10.1074/mcp.R113.029751 23824911 PMC3861702

[42] Tallent MK Varghis N Skorobogatko Y Hernandez-Cuebas L Whelan K Vocadlo DJ In vivo modulation of O-GlcNAc levels regulates hippocampal synaptic plasticity through interplay with phosphorylation. J Biol Chem (2009) 284:174–81. 10.1074/jbc.M807431200 19004831

[43] Francisco H Kollins K Varghis N Vocadlo D Vosseller K Gallo G. O-GLcNAc post-translational modifications regulate the entry of neurons into an axon branching program. Dev Neurobiol (2009) 69:162–73. 10.1002/dneu.20695 19086029 PMC2747243

[44] Skorobogatko Y Landicho A Chalkley RJ Kossenkov AV Gallo G Vosseller K. O-linked beta-N-acetylglucosamine (O-GlcNAc) site thr-87 regulates synapsin I localization to synapses and size of the reserve pool of synaptic vesicles. J Biol Chem (2014) 289:3602–12. 10.1074/jbc.M113.512814 24280219 PMC3916560

[45] Vosseller K Trinidad JC Chalkley RJ Specht CG Thalhammer A Lynn AJ O-linked N-acetylglucosamine proteomics of postsynaptic density preparations using lectin weak affinity chromatography and mass spectrometry. Mol Cell Proteomics (2006) 5:923–34. 10.1074/mcp.T500040-MCP200 16452088

[46] Trinidad JC Barkan DT Gulledge BF Thalhammer A Sali A Schoepfer R Global identification and characterization of both O-GlcNAcylation and phosphorylation at the murine synapse. Mol Cell Proteomics (2012) 11:215–29. 10.1074/mcp.O112.018366 22645316 PMC3412957

[47] Zachara NE O’Donnell N Cheung WD Mercer JJ Marth JD Hart GW. Dynamic O-GlcNAc modification of nucleocytoplasmic proteins in response to stress. A survival response of mammalian cells. J Biol Chem (2004) 279:30133–42. 10.1074/jbc.M403773200 15138254

[48] Slawson C Zachara NE Vosseller K Cheung WD Lane MD Hart GW. Perturbations in O-linked beta-N-acetylglucosamine protein modification cause severe defects in mitotic progression and cytokinesis. J Biol Chem (2005) 280:32944–56. 10.1074/jbc.M503396200 16027160

[49] Banerjee S Robbins PW Samuelson J. Molecular characterization of nucleocytosolic O-GlcNAc transferases of *Giardia lamblia* and *Cryptosporidium parvum*. Glycobiology (2009) 19:331–6. 10.1093/glycob/cwn107 18948359 PMC2733775

[50] Acosta DM Soprano LL Ferrero M Landoni M Esteva MI Couto AS A striking common O-linked N-acetylglucosaminyl moiety between cruzipain and myosin. Parasite Immunol (2011) 33:363–70. 10.1111/j.1365-3024.2011.01291.x 21426361

[51] Perez-Cervera Y Harichaux G Schmidt J Debierre-Grockiego F Dehennaut V Bieker U Direct evidence of O-GlcNAcylation in the apicomplexan *Toxoplasma gondii*: a biochemical and bioinformatic study. Amino Acids (2011) 40:847–56. 10.1007/s00726-010-0702-4 20661758

[52] Uddin N Hoessli DC Butt A Kaleem A Iqbal Z Afzal I O-GlcNAc modification of the anti-malarial vaccine candidate PfAMA1: in silico-defined structural changes and potential to generate a better vaccine. Mol Biol Rep (2012) 39:4663–72. 10.1007/s11033-011-1258-4 22020851

[53] Shen A Kamp HD Grundling A Higgins DE. A bifunctional O-GlcNAc transferase governs flagellar motility through anti-repression. Genes Dev (2006) 20:3283–95. 10.1101/gad.1492606 17158746 PMC1686605

[54] Shi WW Jiang YL Zhu F Yang YH Shao QY Yang HB Structure of a novel O-linked N-acetyl-d-glucosamine (O-GlcNAc) transferase, GtfA, reveals insights into the glycosylation of pneumococcal serine-rich repeat adhesins. J Biol Chem (2014) 289:20898–907. 10.1074/jbc.M114.581934 24936067 PMC4110296

[55] Lewis BA. O-GlcNAcylation at promoters, nutrient sensors, and transcriptional regulation. Biochim Biophys Acta (2013) 1829:1202–6. 10.1016/j.bbagrm.2013.09.003 24076017 PMC3833346

[56] Ranuncolo SM Ghosh S Hanover JA Hart GW Lewis BA. Evidence of the involvement of O-GlcNAc-modified human RNA polymerase II CTD in transcription in vitro and in vivo. J Biol Chem (2012) 287:23549–61. 10.1074/jbc.M111.330910 22605332 PMC3390630

[57] Chen Q Chen Y Bian C Fujiki R Yu X. TET2 promotes histone O-GlcNAcylation during gene transcription. Nature (2013) 493:561–4. 10.1038/nature11742 23222540 PMC3684361

[58] Deplus R Delatte B Schwinn MK Defrance M Mendez J Murphy N TET2 and TET3 regulate GlcNAcylation and H3K4 methylation through OGT and SET1/COMPASS. EMBO J (2013) 32:645–55. 10.1038/emboj.2012.357 23353889 PMC3590984

[59] Shi FT Kim H Lu W He Q Liu D Goodell MA Ten-eleven translocation 1 (Tet1) is regulated by O-linked N-acetylglucosamine transferase (Ogt) for target gene repression in mouse embryonic stem cells. J Biol Chem (2013) 288:20776–84. 10.1074/jbc.M113.460386 23729667 PMC3774349

[60] Zhang Q Liu X Gao W Li P Hou J Li J Differential regulation of the ten-eleven translocation (TET) family of dioxygenases by O-linked beta-N-acetylglucosamine transferase (OGT). J Biol Chem (2014) 289:5986–96. 10.1074/jbc.M113.524140 24394411 PMC3937666

[61] Fujiki R Chikanishi T Hashiba W Ito H Takada I Roeder RG GlcNAcylation of a histone methyltransferase in retinoic-acid-induced granulopoiesis. Nature (2009) 459:455–9. 10.1038/nature07954 19377461

[62] Sakabe K Hart GW. O-GlcNAc transferase regulates mitotic chromatin dynamics. J Biol Chem (2010) 285:34460–8. 10.1074/jbc.M110.158170 20805223 PMC2966060

[63] Sakabe K Wang Z Hart GW. Beta-N-acetylglucosamine (O-GlcNAc) is part of the histone code. Proc Natl Acad Sci U S A (2010) 107:19915–20. 10.1073/pnas.1009023107 21045127 PMC2993388

[64] Bullen JW Balsbaugh JL Chanda D Shabanowitz J Hunt DF Neumann D Cross-talk between two essential nutrient-sensitive enzymes: O-GlcNAc transferase (OGT) and AMP-activated protein kinase (AMPK). J Biol Chem (2014) 289:10592–606. 10.1074/jbc.M113.523068 24563466 PMC4036179

[65] Erickson JR Pereira L Wang L Han G Ferguson A Dao K Diabetic hyperglycaemia activates CaMKII and arrhythmias by O-linked glycosylation. Nature (2013) 502:372–6. 10.1038/nature12537 24077098 PMC3801227

[66] Yi W Clark PM Mason DE Keenan MC Hill C Goddard WA III Phosphofructokinase 1 glycosylation regulates cell growth and metabolism. Science (2012) 337:975–80. 10.1126/science.1222278 22923583 PMC3534962

[67] Tarrant MK Rho HS Xie Z Jiang YL Gross C Culhane JC Regulation of CK2 by phosphorylation and O-GlcNAcylation revealed by semisynthesis. Nat Chem Biol (2012) 8:262–9. 10.1038/nchembio.771 22267120 PMC3288285

[68] Dias WB Cheung WD Hart GW. O-GlcNAcylation of kinases. Biochem Biophys Res Commun (2012) 422:224–8. 10.1016/j.bbrc.2012.04.124 22564745 PMC3387735

[69] Dias WB Cheung WD Wang Z Hart GW. Regulation of calcium/calmodulin-dependent kinase IV by O-GlcNAc modification. J Biol Chem (2009) 284:21327–37. 10.1074/jbc.M109.007310 19506079 PMC2755857

[70] Slawson C Copeland RJ Hart GW. O-GlcNAc signaling: a metabolic link between diabetes and cancer? Trends Biochem Sci (2010) 35:547–55. 10.1016/j.tibs.2010.04.005 20466550 PMC2949529

[71] Lefebvre T Dehennaut V Guinez C Olivier S Drougat L Mir AM Dysregulation of the nutrient/stress sensor O-GlcNAcylation is involved in the etiology of cardiovascular disorders, type-2 diabetes and Alzheimer’s disease. Biochim Biophys Acta (2010) 1800:67–79. 10.1016/j.bbagen.2009.08.008 19732809

[72] Hanover JA Krause MW Love DC. The hexosamine signaling pathway: O-GlcNAc cycling in feast or famine. Biochim Biophys Acta (2010) 1800:80–95. 10.1016/j.bbagen.2009.07.017 19647043 PMC2815088

[73] Ma Z Vosseller K. Cancer metabolism and elevated O-GlcNAc in oncogenic signaling. J Biol Chem (2014) 289 10.1074/jbc.R114.577718 PMC4263853 25336642

[74] Ma Z Vosseller K. O-GlcNAc in cancer biology. Amino Acids (2013) 45:719–33. 10.1007/s00726-013-1543-8 23836420

[75] Shi Y Tomic J Wen F Shaha S Bahlo A Harrison R Aberrant O-GlcNAcylation characterizes chronic lymphocytic leukemia. Leukemia (2010) 24:1588–98. 10.1038/leu.2010.152 20668475 PMC4361888

[76] Lazarus BD Love DC Hanover JA. O-GlcNAc cycling: implications for neurodegenerative disorders. Int J Biochem Cell Biol (2009) 41:2134–46. 10.1016/j.biocel.2009.03.008 19782947 PMC2757632

[77] Yuzwa SA Vocadlo DJ. O-GlcNAc and neurodegeneration: biochemical mechanisms and potential roles in Alzheimer’s disease and beyond. Chem Soc Rev (2014) 43:6839–58. 10.1039/c4cs00038b 24759912

[78] Lagerlof O Hart GW. O-GlcNAcylation of neuronal proteins: roles in neuronal functions and in neurodegeneration. Adv Neurobiol (2014) 9:343–66. 10.1007/978-1-4939-1154-7_16 25151387

[79] Kreppel LK Hart GW. Regulation of a cytosolic and nuclear O-GlcNAc transferase. Role of the tetratricopeptide repeats. J Biol Chem (1999) 274:32015–22. 10.1074/jbc.274.45.32015 10542233

[80] Cole RN Hart GW. Cytosolic O-glycosylation is abundant in nerve terminals. J Neurochem (2001) 79:1080–9. 10.1046/j.1471-4159.2001.00655.x 11739622

